# Diel emergence and dispersal rhythms in the bamboo borer *Dinoderus minutus* (Coleoptera: Bostrichidae)

**DOI:** 10.1007/s00114-026-02181-5

**Published:** 2026-10-08

**Authors:** Felipe Goulart Gonçalves, Hugo Leoncini Rainho, Kamila Emmanuella Xavier de Azevedo, Idemauro Antonio Rodrigues de Lara, Rafael de Andrade Moral, José Maurício Simões Bento

**Affiliations:** 1 Department of Entomology and Acarology, University of São Paulo - USP, Luiz de Queiroz College of Agriculture - ESALQ, Piracicaba, SP 13418-900 Brazil; 2 Instituto Nacional de Coleoptera - INCol, Instituto Nacional de Ciência e Tecnologia - INCT, Cuiabá, Brazil; 3 Department of Exact Sciences, University of São Paulo - USP, Luiz de Queiroz College of Agriculture - ESALQ, Piracicaba, SP 13418-900 Brazil; 4 Department of Mathematics and Statistics, Maynooth University, Maynooth, Ireland

**Keywords:** Behavioral rhythm, Chronobiology, Insect behavior, Powderpost beetle, Wood borer

## Abstract

Biological rhythms regulate insect activity and represent a conserved adaptive trait across life forms. Understanding the behavioral rhythms of dispersive cryptic species is important for elucidating their ecological interactions and improving pest management strategies. In this study, we investigated the diel patterns of emergence and flight activity of the bamboo borer *Dinoderus minutus* under field and laboratory conditions using a chronobiological approach. Field emergence was evaluated using emergence cages containing naturally infested bamboo culms, whereas flight activity was monitored using semiochemical-baited cross-vane panel traps. In laboratory assays, locomotor and flight activities were compared between morning and afternoon periods. Periodicity analyses revealed significant daily rhythms in both emergence and flight activity. Cosinor analyses indicated that most individuals emerged around 14:00 h and initiated flight shortly thereafter, at around 16:00 h, which corresponded to the acrophase of each behavior. No emergence or dispersal activity was recorded during the night period, indicating strictly diurnal dispersal behavior. Laboratory experiments corroborated field observations, showing significantly greater flight initiation, locomotor activity, velocity, and distance traveled during the mid-afternoon than in the morning. These results demonstrate that *D. minutus* exhibits a predictable behavioral periodicity, with peak emergence and flight activity concentrated during the mid-afternoon. This information provides an important basis for field monitoring, detection, and the development of pest management strategies targeting bamboo borer populations.

## Introduction

Chronobiology is a central field in molecular and behavioral biology that investigates the temporal regulation of physiological and behavioral processes across organisms (Dunlap 1999). In insects, biological rhythms regulate essential life-history traits such as feeding, mating, oviposition, metamorphosis, emergence, and dispersal (Wingerden et al. 1992; Stefanescu et al. 2003). These rhythms are controlled by endogenous clocks synchronized by environmental cues, known as zeitgebers, which primarily include light and temperature (Aschoff 1960; Pittendrigh 1960; Dunlap 1999). In some cases, humidity and atmospheric variables may also influence temporal patterns of insect behavior (Pielou and Gunn 1940; Rousse et al. 2009).

The study of behavioral rhythmicity under natural conditions is essential for understanding the ecological organization of insect activity. Most chronobiological studies focus on model organisms or species with conspicuous rhythmic patterns. In contrast, cryptic insects remain poorly investigated, despite their potential to reveal adaptive mechanisms associated with dispersal and survival. One such species is the bamboo borer *Dinoderus minutus* (Fabricius) (Coleoptera: Bostrichidae), a xylophagous insect whose immature stages develop concealed within bamboo culms (Fisher 1950). This cryptic habit protects larvae and pupae from external environmental fluctuations, potentially reducing the influence of abiotic factors on development and survival (Micó 2018). In contrast, adults are exposed to the external environment and display active dispersal behavior.

The protected immature stages and the airborne adult phase may also be associated with reproductive strategies and mate-finding behavior (Levi-Zada and Byers 2021). Under laboratory conditions, *D. minutus* spends its entire immature development and approximately the first week of adult life tunneling inside bamboo material (Watanabe et al. 2017), representing nearly 50% of its lifespan (Garcia and Morrell 2009). Therefore, dispersal represents a critical transition in the adult stage and may be temporally regulated to optimize mating opportunities, host colonization, and avoidance of unfavorable environmental conditions.

Dispersal behavior in insects is frequently synchronized by circadian mechanisms and may be influenced by abiotic factors (Chen and Seybold 2014). In this study, we investigated the diel emergence and flight activity of *D. minutus* under field and laboratory conditions to evaluate the temporal organization of its dispersal behavior.

## Materials and methods

### Insect rearing

*Dinoderus minutus* rearing was established using cut culms of bamboo (Poaceae: Bambusoideae), *Bambusa vulgaris* Schrad. At the beginning of the autumn season, bamboo culms were exposed in the field to allow colonization by adult beetles in a shaded area located near remnants of the Atlantic Rainforest (Piracicaba, Brazil: 22°42′45″ S, 47°37′41″ W; 540 m above sea level), where both native vegetation and exotic bamboo species, including *B. vulgaris* and *Dendrocalamus giganteus* Munro, were present.

The bamboo samples (30 cm × 7 cm in diameter) presented approximately 15% moisture content when exposed in the field, providing suitable conditions for beetle infestation (Garcia and Morrell 2009). To ensure the collection of the first generation of *D. minutus* emerging from the infested culms, the bamboo material was transferred to the laboratory approximately one month after the first detection of adult boring holes. The culms were then maintained inside emergence cages designed according to the principles proposed by Saunders and Knoke (1967) for studies involving xylophagous beetles.

The emergence cages consisted of black plastic containers (50 × 32 × 32 cm) containing a single opening that functioned as the only light entrance. A translucent collecting bottle was attached to this opening, serving as the sole light-attractive point within the cage. At least one emergence cage was maintained under field conditions and another under laboratory conditions.

Laboratory colonies were maintained in a climate-controlled room at 25 ± 1 °C and 60 ± 10% relative humidity under an LD 12:12 h photoperiod, with lights on at 06:00 h and off at 18:00 h. This regime was the standard condition used to maintain and synchronize the laboratory colony and was not intended to reproduce the exact natural photoperiod during the field experiment.

### Data collection

#### Field emergence experiment

The field emergence experiment with adult *D. minutus* was conducted by maintaining the emergence cage outdoors, under natural meteorological conditions, while protected from direct sunlight and rainfall. Adult emergence was monitored daily from 06:00 to 20:00 h. Beetles were identified and counted every 2 h by inspecting the translucent collection bottle attached to the cage. Emergence occurring during the night period was also recorded. After each evaluation interval, the collection bottle was emptied to avoid recounting individuals. The experiment was conducted over a 14-day period, from 18 June to 10 July 2020, totaling 10 effective observation days. Days without beetle emergence were excluded from the analyses.

#### Field flight experiment

The daily flight periodicity of adult *D. minutus* was investigated using translucent cross-vane panel traps baited with a semiochemical blend composed of hexanal and decanal, previously demonstrated to attract both males and females of the species (Rainho et al. 2022). Four traps were installed in a linear transect within the same study area described previously. The traps were suspended from inverted L-shaped PVC pipe hangers (1.8 m height, 2.5 cm internal diameter), with the trap panels positioned approximately 1.2 m above ground level. Collection bottles attached to the trap bases were filled with 250 mL of a 20% aqueous propylene glycol solution to kill and preserve captured insects. Collections were conducted daily from 06:00 to 20:00 h. Captured beetles were identified and counted every 2 h by examining the collection bottles. Total overnight captures were also recorded. After each evaluation interval, the collection bottles were emptied to avoid recounting individuals. The experiment was conducted simultaneously with the field emergence experiment and comprised 16 consecutive collection days, from 18 June to 10 July 2020. Days without beetle captures were excluded from the analyses.

#### Laboratory activity experiment

Thirty newly emerged adults of *D. minutus* obtained from the laboratory colony were individually transferred to Petri dishes (15 cm diameter). Each adult constituted one experimental unit and was evaluated at 10:00 h and 16:00 h, representing morning and afternoon conditions, respectively (*n* = 30 observations at each time point). At each time point, insects were recorded for 30 min. Flight behavior was evaluated by recording the number of flight attempts by each insect and whether flight initiation occurred. Locomotor activity was quantified from the videos using EthoVision software (Noldus et al. 2001), which measured the distance traveled and mean movement velocity during the observation period.

### Statistical analyses

Laboratory data were analyzed according to response type. The occurrence of flight initiation was analyzed using a binomial GLM with time of day (10:00 or 16:00 h) as the explanatory factor. The number of flight attempts was analyzed using a Poisson GLM and, when overdispersion was detected, a quasi-Poisson model. Distance traveled and mean velocity were analyzed using Gaussian linear models with time of day as the explanatory factor. Model significance was assessed using likelihood-ratio tests for binomial and Poisson models and F-tests for quasi-Poisson and Gaussian models.

The periodicity of both emergence and flight activity was evaluated using Lomb–Scargle Periodogram (LSP) analyses (Lomb 1976; Scargle 1982). For discrete response variables, including the number of flight events and captured or emerged *D. minutus* adults, Generalized Linear Models (GLMs) (Wedderburn 1974) and their extensions were employed. In these models, the linear predictor was specified as response variable ~ observation type + time of day, where observation type was included as a categorical factor with two levels (flight and emergence), and time of day was included as a categorical factor, with each observation time treated as a separate level. Count data were initially fitted using Poisson GLMs, whereas proportion data were analyzed using binomial GLMs. Model significance was assessed through likelihood-ratio (LR) tests comparing nested models.

Whenever overdispersion violated the mean–variance assumptions of the models, quasi-likelihood approaches were adopted. Acrophase estimates were obtained using Cosinor models (Parsons et al. 2024), assuming a 24 h periodicity. In contrast to the conventional GLMs, time of day was not included as a categorical factor in the cosinor models, but was incorporated into the periodic component of the model using cumulative time as the temporal predictor for both emergence and flight datasets. Observation type was retained as a grouping predictor, allowing the periodic patterns of emergence and flight activity to be estimated and compared within the same model framework. Differences between the estimated rhythmic components were assessed using Wald tests.

All statistical analyses and graphical representations were performed in R (R Core Team 2024) using the packages *lme4* (Bates et al. 2015) for model fitting, *hnp* (Moral et al. 2017) for GLM goodness-of-fit diagnostics, *GLMMcosinor* (Parsons et al. 2024) for Cosinor analyses, and *lomb* (Ruf 1999) for Lomb–Scargle Periodogram analyses. Figures were generated using the package *ggplot2* (Wickham 2011).

## Results

Both emergence and flight activity of *D. minutus* exhibited significant daily periodicity under field conditions (LSP, Emergence: *per* = 23.909, *p* < 0.01; Flight: *per* = 23.846, *p* < 0.01). The highest proportion of emerging adults was recorded at 14:00 h, accounting for approximately 65% of the total emergence, whereas peak flight activity occurred at 16:00 h, representing nearly 60% of total captures in the field.

**Fig. 1:**
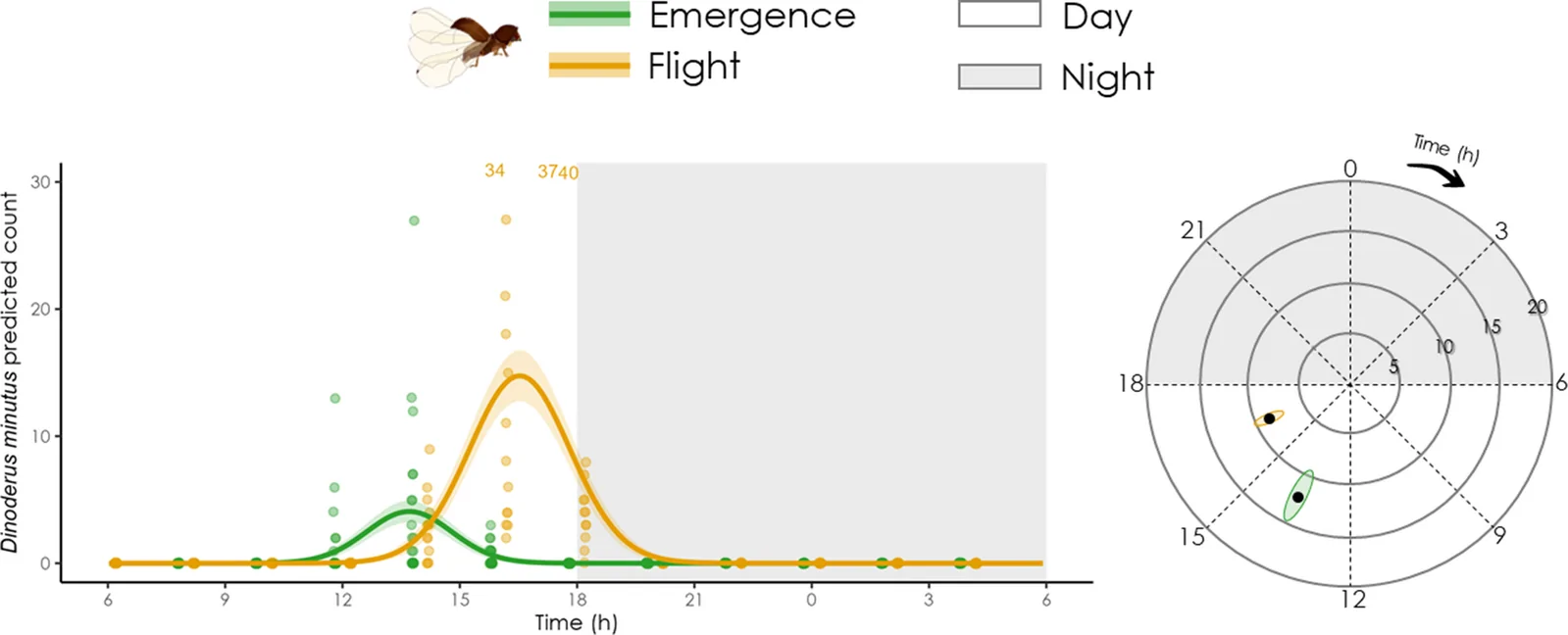
Predicted diel patterns of *Dinoderus minutus* activity obtained from Poisson cosinor generalized linear models. Solid lines represent model-predicted mean counts across the 24-h cycle for emergence (green; 10 observation days) and flight (yellow; 16 observation days), with shaded areas indicating 95% confidence intervals. Points represent counts observed at each 2-h sampling interval. Numbers above the curves (34, 37, and 40) are observed counts that exceeded the upper limit of the y-axis and are printed at their corresponding sampling times. Points for emergence and flight were slightly offset horizontally to prevent overlap. The gray background represents the night period, whereas the white background represents the day period. The polar plot (right) illustrates the estimated acrophase and amplitude for each activity, with confidence regions derived from Wald elliptical tests

Cosinor analyses indicated significant 24-h periodic components in both emergence and flight activity. The estimated acrophase occurred at approximately 13:42 h for emergence (acrophase = − 2.70 rad, SE = 0.03, 95% CI: −2.75 to − 2.64, *p* < 0.001) and 16:32 h for flight activity (acrophase = − 1.96 rad, SE = 0.02, 95% CI: −1.99 to − 1.92, *p* < 0.001) (Fig. 1). The acrophases differed significantly between emergence and flight activity (Wald statistic = 22.22, *p* < 0.001), with an estimated phase difference of 0.74 rad (95% CI: 0.67–0.81), corresponding to approximately 2.83 h.

The estimated amplitudes were significantly different from zero for both emergence (12.33, SE = 1.44, 95% CI: 9.50–15.15, *p* < 0.001) and flight activity (8.87, SE = 0.69, 95% CI: 7.52–10.22, *p* < 0.001). Amplitude also differed significantly between the two behaviors (Wald statistic = − 2.17, *p* = 0.030), with flight activity showing a lower amplitude than emergence (difference = − 3.46, 95% CI: −6.59 to − 0.33). These results support distinct diel patterns for emergence and flight activity. No dispersal activity was observed during the overnight period.

Laboratory behavioral assays corroborated the field observations and demonstrated a marked increase in flight-related activity during the afternoon period.

**Fig. 2:**
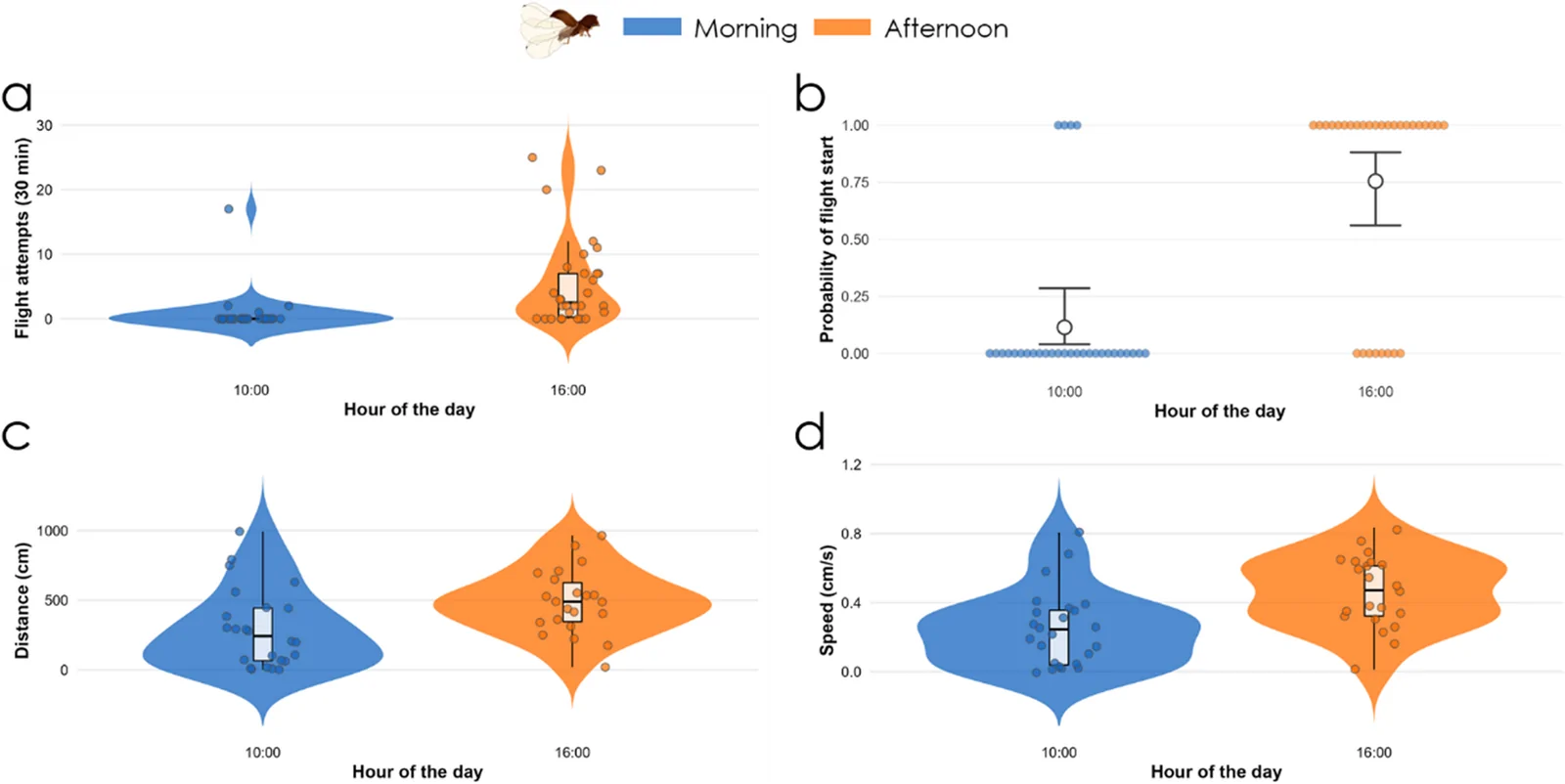
Flight behavior (**a**, **b**) and locomotor activity (**c**, **d**) of *Dinoderus minutus* recorded in Petri dishes during 30-min observation periods (*n* = 30 individual beetles and 30 observations per time point). Experiments were conducted at 10:00 h and 16:00 h. (**a**) Number of flight attempts, (**b**) probability of flight initiation, (**c**) distance traveled, and (**d**) mean velocity. Violin plots represent data distributions, dots indicate individual observations, and boxplots show medians and interquartile ranges. In panel (**b**), confidence intervals correspond to estimated marginal means on the response scale. All variables differed significantly between 10:00 h and 16:00 h (*p* < 0.05)

 Adults of *D. minutus* evaluated at 16:00 h exhibited significantly more flight attempts than those evaluated at 10:00 h (quasi-Poisson GLM, F = 10.963, df = 1, *p* < 0.01) (Fig. 2a). Likewise, the probability of flight initiation was significantly higher at 16:00 h (binomial GLM, χ^2^ =23.72, df = 1, *p* < 0.01) (Fig. 2b).

In addition to the higher frequency of flight initiation, beetles evaluated in the afternoon also displayed increased locomotor performance. Flight speed was significantly higher at 16:00 h compared to 10:00 h (One-way ANOVA, F(1,29) = 11.614, *p* < 0.01) (Fig. 2c). Likewise, traveled distance was significantly greater during the afternoon evaluations (One-way ANOVA, F(1,29) = 6.568, *p* < 0.05) (Fig. 2d). Together, these results indicate that both dispersal-related flight behavior and general locomotor activity *of D. minutus* are strongly synchronized with a daily pattern, with peak activity concentrated during the afternoon period.

## Discussion

Field and laboratory observations consistently showed a diel pattern in *D. minutus* emergence and dispersal-related activity. Under field conditions, adult emergence peaked near 14:00 h and flight activity near 16:00 h, whereas laboratory activity was greater at 16:00 h than at 10:00 h. These results establish the timing of activity under the environmental cycles tested, but they do not demonstrate control by an endogenous circadian clock.

Adults maintained under laboratory conditions showed greater locomotor and flight activity at 16:00 h than at 10:00 h. Because the assay compared only two time points within an LD cycle on a single day, it demonstrates a time-of-day difference rather than free-running rhythmicity. Testing endogenous circadian control would require continuous observations over multiple cycles under constant environmental conditions, because circadian rhythmicity is generated and maintained by molecular feedback mechanisms involving clock genes (Giebultowicz 2001; Saunders 2002; Hardin 2011).

Similar temporal activity patterns have been reported for other terrestrial insect species. The Asian citrus psyllid, *Diaphorina citri* Kuwayama, exhibits increased dispersal activity during the afternoon period (Tomaseto et al. 2018). Likewise, black flies (Davies and Roberts 1980) and the Asian gypsy moth, *Lymantria dispar* L. (Cardé et al. 1996), also display behavioral peaks near 16:00 h. More recently, Kirse et al. (2025), using continuous multispecies insect sampling over an entire year, demonstrated that several insect groups exhibit maximum activity during the mid-afternoon, with Coleoptera showing particularly pronounced peaks around 16:00 h.

Among beetles with cryptic or endophytic habits, similar chronobiological patterns have also been documented. The coffee berry borer, *Hypothenemus hampei* (Ferrari) (Curculionidae: Scolytinae), for example, exhibits late-afternoon sexual activity under laboratory conditions (Silva et al. 2012) and a marked mid-afternoon dispersal peak under field conditions (Baker et al. 1992). The similarities between *H. hampei* and *D. minutus* suggest that synchronized afternoon dispersal may represent an adaptive strategy in concealed wood- or seed-boring beetles, potentially optimizing mate finding, host colonization, or environmental conditions favorable for flight.

The cues synchronizing emergence inside bamboo remain unresolved. In insects, diel activity patterns can arise from interactions between endogenous timing mechanisms and environmental or ecological factors, including rhythmic gene expression, hormonal regulation, light sensitivity, thermoregulation, predator avoidance, competition, and resource availability (Bakken 1992; Heinrich 1993; Shimizu and Barth 1996; Giebultowicz 2001; Saunders 2002; Hardin 2011; Warrant and Dicke 2011). Light entering through adult exit holes or thin and damaged regions of the culm could provide a photic cue, although light transmission through intact bamboo walls and light perception by concealed pupae were not measured. Daily temperature fluctuations may also propagate through the culm and contribute to synchronization, potentially together with light. However, neither temperature inside the culms nor light penetration was quantified in this study, so their relative roles cannot be distinguished.

A further limitation is that temperature was not recorded at the experimental site. Data from a regional weather station were not added because they would not reliably represent the shaded microclimates of the emergence cage, bamboo culms, and flight traps. Consequently, the observed afternoon peaks cannot be attributed specifically to temperature, the light-dark cycle, or their interaction. Simultaneous microclimatic measurements and constant-condition experiments will be needed to identify the proximate cues and determine whether an endogenous component is present.

The close temporal sequence between emergence and flight is ecologically relevant because newly emerged adults can rapidly leave the natal substrate and begin host-searching. Concentrating monitoring and behavioral observations in the mid-afternoon should therefore increase the probability of detecting dispersing adults.

*Dinoderus minutus* represents an extreme example of host specialization, colonizing primarily injured culms of a limited number of bamboo genera and responding to specific blends of straight-chain aldehydes emitted by damaged host plants (Garcia and Morrell 2009; Rainho et al. 2022). In this context, the synchronization of dispersal and host colonization during the mid-afternoon may represent an adaptive mechanism associated with the limited temporal and spatial availability of suitable host substrates. In addition, temporal specialization may contribute to reducing interspecific competition with other bamboo-associated insects occupying similar ecological niches within its native distribution range.

Our results show that the bamboo borer exhibits a repeatable diel activity pattern under the tested field conditions, with adult emergence peaking around 14:00 h and flight activity peaking later in the afternoon. These findings clarify the temporal organization of dispersal behavior without assigning it to a specific environmental cue or endogenous mechanism. The identified activity window also defines an appropriate period for field detection, behavioral observations, and studies of flight and host colonization.

From an applied perspective, understanding the temporal dynamics of *D. minutus* activity may contribute to the development of more efficient management strategies for bamboo borer control. Knowledge of the daily timing of emergence and dispersal can support the optimization of monitoring programs, improve the timing of semiochemical-based trapping systems, and assist in the protection of freshly injured bamboo culms, which represent the most susceptible substrates for colonization.

## Data Availability

Behavioral data and analysis codes of each experiment are present on Figshare repositories at DOI: https://doi.org/10.6084/m9.figshare.31915395.

## References

[CR1] Aschoff J (1960) Exogenous and endogenous components in circadian rhythms. Cold Spring Harb Symp Quant Biol 25:11–28. 10.1101/SQB.1960.025.01.004 13684695

[CR2] Baker PS, Ley C, Balbuena R, Barrera JF (1992) Factors affecting the emergence of *Hypothenemus hampei* (Coleoptera: Scolytidae) from coffee berries. Bull Entomol Res 82(2):145–150. 10.1017/S0007485300051655

[CR3] Bakken GS (1992) Measurement and application of operative and standard operative temperatures in ecology. Am Zool 32(2):194–216. 10.1093/icb/32.2.194

[CR4] Bates D, Maechler M, Bolker B, Walker S, Christensen RHB, Singmann H, Bolker MB (2015) Package ‘lme4’. Convergence 12(1):2. 10.18637/jss.v067.i01

[CR5] Cardé RT, Charlton RE, Wallner WE, Baranchikov YN (1996) Pheromone-mediated diel activity rhythms of male Asian gypsy moths (Lepidoptera: Lymantriidae) in relation to female eclosion and temperature. Ann Entomol Soc Am 89(5):745–753. 10.1093/aesa/89.5.745

[CR6] Chen Y, Seybold SJ (2014) Crepuscular flight activity of an invasive insect governed by interacting abiotic factors. PLoS One 9(8):e105945. 10.1371/journal.pone.0105945 PMC4144967 25157977

[CR7] Davies L, Roberts DM (1980) Flight activity of female black-flies (Diptera: Simuliidae) studied with a vehicle-mounted net in northern England. J Nat Hist 14(1):1–16. 10.1080/00222938000770011

[CR8] Dunlap JC (1999) Molecular bases for circadian clocks. Cell 96(2):271–290. 10.1016/S0092-8674(00)80566-8 9988221

[CR9] Fisher WS (1950) A revision of the North American species of beetles belonging to the family Bostrichidae (No. 698). US Government Printing Office

[CR10] Garcia CM, Morrell JJ (2009) Development of the powderpost beetle (Coleoptera: Bostrichidae) at constant temperatures. Environ Entomol 38(2):478–483. 10.1603/022.038.0224 19389298

[CR11] Giebultowicz JM (2001) Peripheral clocks and their role in circadian timing: insights from insects. Philosophical Trans Royal Soc B: Biol Sci 356(1415):1761–1769. 10.1098/rstb.2001.0963 PMC1088555 11710986

[CR12] Hardin PE (2011) Molecular genetic analysis of circadian timekeeping in *Drosophila*. Adv Genet 74:141–173. 10.1016/B978-0-12-387690-4.00005-2 PMC4108082 21924977

[CR13] Heinrich B (1993) The Hot-Blooded Insects: Strategies and Mechanisms of Thermoregulation. Harvard University Press

[CR14] Kirse A, Wittenhorst MA, Scherber C, Posanski M, Scherges A, Zizka V, Ott D, Noll NW, Wägele WJ (2025) The clockwork of insect activity: advancing ecological understanding through automation. J Anim Ecol. 10.1111/1365-2656.14246 PMC11962232 39853776

[CR15] Levi-Zada A, Byers JA (2021) Circadian rhythms of insect pheromone titer, calling, emission, and response: a review. Sci Nat 108(5):35. 10.1007/s00114-021-01741-1 34423384

[CR16] Lomb NR (1976) Least-squares frequency analysis of unequally spaced data. Astrophys Space Sci 39:447–462

[CR17] Micó E (2018) Saproxylic insects in tree hollows. Saproxylic insects: diversity, ecology and conservation, 693–727. 10.1007/978-3-319-75937-1_21

[CR18] Moral RA, Hinde J, Demétrio CGB (2017) Half-normal plots and overdispersed models in R: the hnp package. J Stat Softw 81(10). 10.18637/jss.v081.i10

[CR19] Noldus LP, Spink AJ, Tegelenbosch RA (2001) EthoVision: a versatile video tracking system for automation of behavioral experiments. Behav Res Methods Instruments Computers 33:398–414. 10.3758/BF03195394 11591072

[CR20] Parsons R, Jayasinghe O, White N, Chunduri P, Rawashdeh O (2024) GLMMcosinor: Flexible cosinor modeling with a generalized linear mixed modeling framework to characterize rhythmic time series. bioRxiv, 2024-04. 10.1101/2024.04.10.588934

[CR21] Pielou DP, Gunn DL (1940) The humidity behaviour of the mealworm beetle, *Tenebrio molitor* L: I. The reaction to differences of humidity. J Exp Biol 17(3):286–294

[CR22] Pittendrigh CS (1960) Circadian rhythms and the circadian organization of living systems. Cold Spring Harb Symp Quant Biol 25:159–184. 10.1101/SQB.1960.025.01.015 13736116

[CR23] R Core Team (2024) R: A Language and Environment for Statistical Computing. R Foundation for Statistical Computing, Vienna, Austria. https://www.R-project.org

[CR24] Rainho HL, Silva WD, Gonçalves FG, Savaris M, Bento JMS (2022) Hexanal combined with decanal mediate host location by the bamboo powderpost beetle, *Dinoderus minutus*. Entomol Exp Appl 170(9):805–811. 10.1111/eea.13205

[CR25] Rousse P, Gourdon F, Roubaud M, Chiroleu F, Quilici S (2009) Biotic and abiotic factors affecting the flight activity of *Fopius arisanus*, an egg-pupal parasitoid of fruit fly pests. Environ Entomol 38(3):896–903. 10.1603/022.038.0343 19508801

[CR26] Ruf T (1999) The Lomb-Scargle periodogram in biological rhythm research: analysis of incomplete and unequally spaced time-series. Biol Rhythm Res 30(2):178–201. 10.1076/brhm.30.2.178.1422 11708361

[CR27] Saunders DS (2002) Insect Clocks. Elsevier

[CR28] Saunders JL, Knoke JK (1967) Diurnal emergence of *Xyleborus ferrugineus* (Coleoptera: Scolytidae) from cacao trunks in Ecuador and Costa Rica. Ann Entomol Soc Am 60(5):1094–1096. 10.1093/aesa/60.5.1094

[CR29] Scargle JD (1982) Studies in astronomical time series analysis. II-Statistical aspects of spectral analysis of unevenly spaced data. Astrophysical Journal, Part 1 263:835–853

[CR30] Shimizu I, Barth FG (1996) Light-dependent modulation of insect behavior. J Comp Physiol A 179(4):443–458. 10.1007/BF00194996

[CR31] Silva WD, Mascarin GM, Romagnoli EM, Bento JMS (2012) Mating behavior of the coffee berry borer, *Hypothenemus hampei* (Ferrari) (Coleoptera: Curculionidae: Scolytinae). J Insect Behav 25(4):408–417. 10.1007/s10905-012-9313-0

[CR32] Stefanescu C, Peñuelas J, Filella I (2003) Effects of climatic change on the phenology of butterflies in the northwest Mediterranean Basin. Glob Change Biol 9(10):1494–1506. 10.1046/j.1365-2486.2003.00682.x

[CR33] Tomaseto AF, Miranda MP, Moral RA, de Lara IA, Fereres A, Lopes JRS (2018) Environmental conditions for *Diaphorina citri* Kuwayama (Hemiptera: Liviidae) take-off. J Appl Entomol 142(1–2):104–113. 10.1111/jen.12413

[CR34] Warrant E, Dicke M (2011) Vision and visual navigation in nocturnal insects. Ann Rev Entomol 56:239–254. 10.1146/annurev-ento-120709-144840 20822443

[CR35] Watanabe H, Yanase Y, Fujii Y (2017) Nondestructive evaluation of egg-to-adult development and feeding behavior of the bamboo powderpost beetle *Dinoderus minutus* using X-ray computed tomography. J Wood Sci 63(5):506–513. 10.1007/s10086-017-1642-3

[CR36] Wedderburn RWM (1974) Generalized linear models specified in terms of constraints. J Roy Stat Soc: Ser B (Methodol) 36(3):449–454

[CR37] Wickham H (2011) ggplot2. Wiley interdisciplinary reviews: Comput Stat 3(2):180–185

[CR38] Wingerden WV, Kreveld AV, Bongers W (1992) Analysis of species composition and abundance of grasshoppers (Orth., Acrididae) in natural and fertilized grasslands. J Appl Entomol 113(1–5):138–152. 10.1111/j.1439-0418.1992.tb00644.x

